# The first mitochondrial genome of *Codonobdella* sp. (Hirudinea, piscicolidae), an endemic leech species from Lake Baikal, Russia and reassembly of the *Piscicola geometra* data from SRA

**DOI:** 10.1080/23802359.2021.1967807

**Published:** 2021-10-05

**Authors:** Alexander Bolbat, Ekaterina Matveenko, Elena Dzyuba, Irina Kaygorodova

**Affiliations:** Limnological Institute of Siberian Branch of the Russian Academy of Sciences, Irkutsk, Russia

**Keywords:** Complete mitochondrial genome, *Codonobdella* sp., *Piscicola geometra*, Piscicolidae, Hirudinea

## Abstract

In this study, we assembled a complete mitochondrial genome of *Codonobdella* sp. sample from Lake Baikal, Russia and reassembled third party raw data of *Piscicola geometra*. Mitogenomes of both freshwater piscine leeches consist of 37 genes, including 13 protein-coding genes (PCGs), two ribosomal genes (12S and 16S), 22 transport RNA genes, and one control region. The complete mitogenome of *Codonobdella* sp. is 14,486 bp long and A + T biased (75.51%). The complete mitogenome of *Piscicola geometra* is 14,788 bp long and A + T biased (78.27%).

The *Codonobdella* sp. is a potentially new species of abyssal piscine leeches belonging to the genus endemic to Lake Baikal, which, according to V. Epstein (1987), was represented by the only species *C. truncata* Grube, 1873, morphologically and ecologically different from the studied sample. *Codonobdella* sp. was primary found by I. Kaygorodova (2012) on amphipods throughout the lake on depth 40–860 m. It differs from the *C. truncata* at least by existence of a distinctive pigmentation on the dorsal side, size and shape of body. The further morphological analysis is necessary to describe the new species. Decoding the complete mitochondrial genome may serve as a foundation for evolutionary investigations. The study of genomic sequences may shed light on nuances of molecular evolution mechanisms as well as conditions of endemic species existence and survival.

The samples of *Codonobdella* sp. were taken from fishing nets in the Maloe More Strait of Lake Baikal, Russia: 53°15'36.9"N 107°06'45.7"E. Biological material is deposited in Limnological Institute, Russia (contact person Dr. Irina Kaygorodova, irina@lin.irk.ru) under voucher no. B45. The total DNA was extracted with the DiaGene Cell Culture kit. The reads were obtained through Illumina NextSeq 550 at the IC&G Center of Genomic Investigations (Novosibirsk, Russia). Due to incompleteness of the *Piscicola geometra* (L., 1761) MT628553 assembly, we used raw SRR12518725 reads from the Short Read Achieve (SRA) to reassemble its mitogenome. This widespread freshwater species is the closest Piscicolidae relative, available in public sources. The assembly of both samples was performed in MIRA5 (Chevreux et al. 1999). Protein annotation was carried out by BLASTp search of translated open reading frames. Transfer RNAs were predicted using ARAGORN (Laslett and Canback 2004).

The complete mitogenomes of *Codonobdella* sp. and *Piscicola geometra* are 14,486 and 14,788 bp long with the AT content of 75.51% and 78.27%, correspondingly. A total of 37 genes were identified on both mitogenome sequences, including 13 PCGs, two ribosomal genes (12S and 16S), 22 transport RNA genes, and one putative control region. The genes order is the same as for marine leech *Zeylanicobdella arugamensis* de Silva, 1963 (KY474378), the only complete mitogenome of the Piscicolidae family available in GenBank prior to this study.

Phylogenetic position of the first obtained mitogenome sequence of *Codonobdella* sp. relative to 57 evolutionarily close taxa (Oligochaeta, Hirudinea and Polychaeta) available in the NCBI database was inferred using BEAST v2.6.0 (Bouckaert et al. 2019) under optimal substitution model (GTR + G + I) selected by jModelTest v2.1.10 (Darriba et al. 2012). The resulting tree (Figure 1) showed that all samples grouped into distinct clades according to their taxonomy. The *Codonobdella* sp. and *P. geometra* sequences, together with that of *Z. arugamensis*, formed the clade of the Piscicolidae representatives within the lineage of Rhynchobdellida. This is fully consistent with the modern classification of leech taxa.

**Figure 1.**
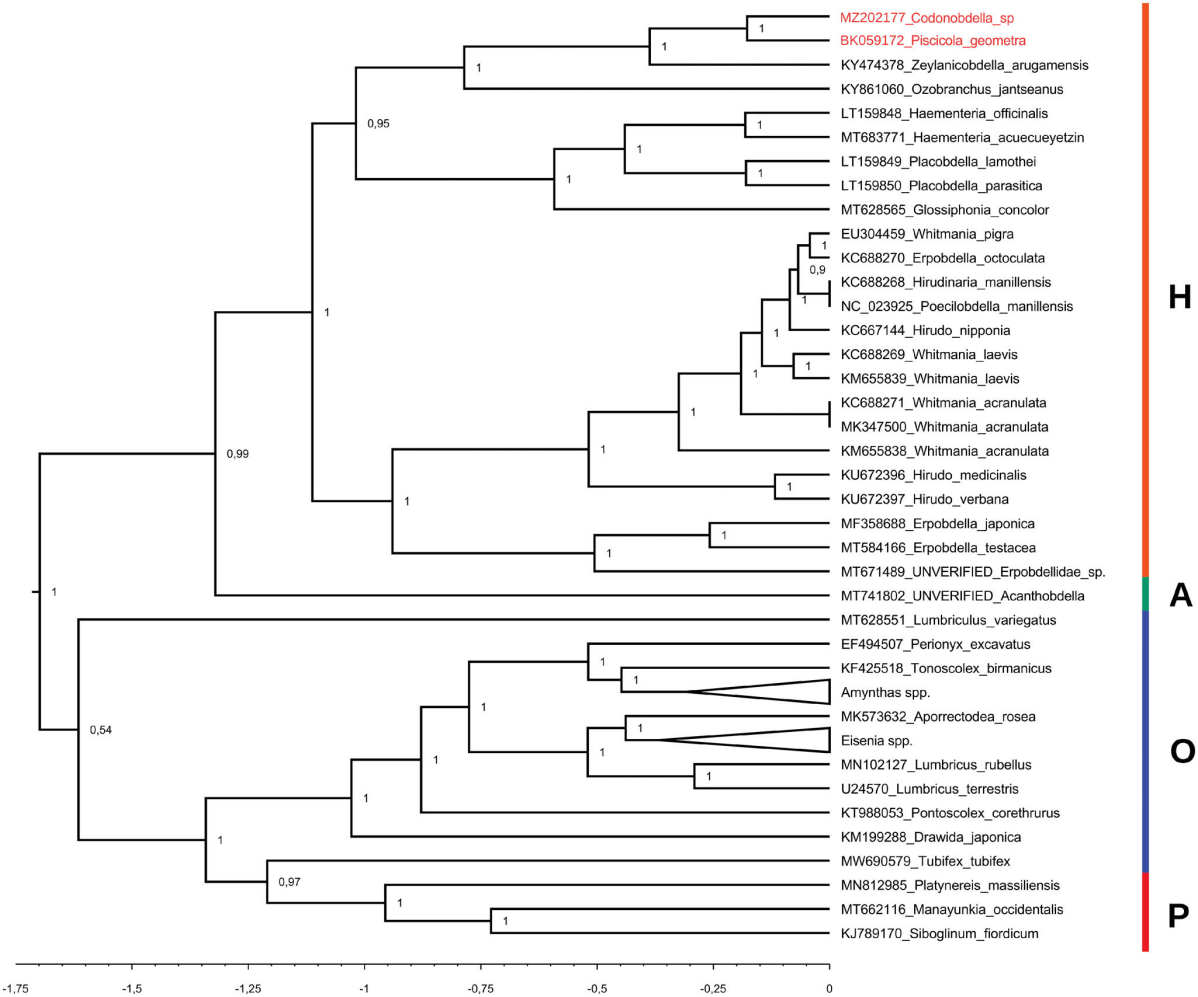
Bayesian phylogenetic tree with posterior probability values. The scale axis below represents time in proportion of substitutions. Clades of Polychaeta (P), Oligochaeta (O), Acanthobdellida (A) and Hirudinea (H) are highlighted in different colors.

## Data Availability

The original data of this study have open access at https://www.ncbi.nlm.nih.gov/, reference number MZ202177. The associated BioProject is accessible at https://www.ncbi.nlm.nih.gov/sra/PRJNA733851, with SRA SRR14709013 and BioSample SAMN19460315. The Third Party Annotation is deposited at the DDBJ/ENA/GenBank databases under the accession number TPA: BK059172.
